# Transcriptome Analysis Reveals High Similarities between Adult Human Cardiac Stem Cells and Neural Crest-Derived Stem Cells

**DOI:** 10.3390/biology9120435

**Published:** 2020-12-01

**Authors:** Anna L. Höving, Katharina Sielemann, Johannes F. W. Greiner, Barbara Kaltschmidt, Cornelius Knabbe, Christian Kaltschmidt

**Affiliations:** 1 Department of Cell Biology, Bielefeld University, 33615 Bielefeld, Germany; johannes.greiner@uni-bielefeld.de (J.F.W.G.); barbara.kaltschmidt@uni-bielefeld.de (B.K.); 2 Heart and Diabetes Centre NRW, Institute for Laboratory and Transfusion Medicine, Ruhr-University Bochum, 32545 Bad Oeynhausen, Germany; cknabbe@hdz-nrw.de; 3 Genetics and Genomics of Plants, Center for Biotechnology (CeBiTec), Bielefeld University, 33615 Bielefeld, Germany; kfrey@cebitec.uni-bielefeld.de; 4 Graduate School DILS, Bielefeld Institute for Bioinformatics Infrastructure (BIBI), Bielefeld University, 33615 Bielefeld, Germany; 5 AG Molecular Neurobiology, Bielefeld University, 33615 Bielefeld, Germany

**Keywords:** adult human stem cells, cardiac stem cells, neural crest-derived inferior turbinate stem cells, stem cell niche, RNA-Seq, transcriptome analysis

## Abstract

**Simple Summary:**

The regeneration of nearly all organs of the human body mainly depends on the functionality of adult stem cell populations that reside in their respective niches and can be activated upon injuries or other damages. These stem cell populations greatly differ in their expression profile of molecular markers, which greatly influences their potential use in regenerative medicine. Neural crest-derived stem cells are a prominent subpopulation of adult stem cells and are known for their high regenerative potential. Within this study, we compared two adult human stem cell populations, namely neural crest-derived inferior turbinate stem cells from the nasal cavity and human cardiac stem cells from the heart, using global gene expression profiling. Here, we found differences that correspond to the tissue sources of origin but also similarities in the expression of markers that are associated with the neural crest. Further classifying nasal stem cells and cardiac stem cells in a broader context, we identified clear similarities between both populations and other adherent stem cell populations compared to non-adherent progenitor cells of the blood system. The analyses provided here might help to understand the differences and similarities between different adult human stem cell populations.

**Abstract:**

For the identification of a stem cell population, the comparison of transcriptome data enables the simultaneous analysis of tens of thousands of molecular markers and thus enables the precise distinction of even closely related populations. Here, we utilized global gene expression profiling to compare two adult human stem cell populations, namely neural crest-derived inferior turbinate stem cells (ITSCs) of the nasal cavity and human cardiac stem cells (hCSCs) from the heart auricle. We detected high similarities between the transcriptomes of both stem cell populations, particularly including a range of neural crest-associated genes. However, global gene expression likewise reflected differences between the stem cell populations with regard to their niches of origin. In a broader analysis, we further identified clear similarities between ITSCs, hCSCs and other adherent stem cell populations compared to non-adherent hematopoietic progenitor cells. In summary, our observations reveal high similarities between adult human cardiac stem cells and neural crest-derived stem cells from the nasal cavity, which include a shared relation to the neural crest. The analyses provided here may help to understand underlying molecular regulators determining differences between adult human stem cell populations.

## 1. Introduction

Adult human stem cell (ASC) populations harbour a great potential for applications in regenerative medicine [1,2], emphasizing the importance of their identification, characterization and classification. In recent years, ASCs were described in nearly all tissues and organs of the human body [3,4,5,6,7,8,9]. However, these populations showed strong differences in their potential to differentiate into specialized cell types. Although most of these differences in differentiation potential were linked to developmental origin and the tissue of origin of the respective stem cell population, some tissues harbour multiple stem cell populations with highly different potentials. For instance, populations of skeletal stem cells could be found in the bone marrow next to hematopoietic stem cells and mesenchymal stem cells [10,11]. Moreover, mesenchymal stem cells with different functionalities were found in diverse tissues [12,13]. For instance, Maleki and coworkers showed that spermatogonial stem cells and Wharton’s jelly-mesenchymal stem cells are all able to differentiate into the osteogenic lineage [13]. However, Riekstina and colleagues showed that adult mesenchymal stem cell populations, derived from bone marrow, adipose tissue, dermis and the heart, express different combinations of stem cell markers in vitro [12]. With regard to these differences, defining adult stem cell populations still remains challenging. On a technical level, analysis of cell surface proteins accompanied by laborious differentiation assays were commonly applied to characterize human stem cells pools [13,14,15,16]. Although this method led to the identification of a large number of cell populations, the classification of stem cell pools by marker proteins was mostly limited to the number of available fluorochromes or filters that could be used simultaneously. Addressing this challenge, the analysis of RNA sequencing (RNA-Seq) data is a widely used and powerful tool allowing the simultaneous comparison and quantification of hundreds of biomarkers and underlying signaling pathways contributing to the varying stem cell phenotypes. RNA-Seq also enables the determination of expression profiles and pathways conserved between stem cell populations from different sources, thus allowing a more precise characterization and classification.

In the present study, we used RNA-Seq to characterize and compare a very recently identified Nestin^+^/CD105^+^ adult human cardiac stem cell population (hCSCs) [17] with neural crest-derived stem cells from the inferior turbinate of the human nose (inferior turbinate stem cells, ITSCs) [8]. Although the human heart was initially considered as a terminal differentiation organ, several populations of adult cardiac stem cells were shown to reside within the human heart. These cardiac stem cells were shown to differentiate into cardiomyocytes as well as into smooth muscle cells and endothelial cells in vitro [18,19,20]. We very recently extended these findings by identifying a Nestin^+^/CD105^+^ adult hCSC population that could be isolated from the left atrial appendage (LAA) and that gave rise to cardiomyocytes in vitro [17]. Nevertheless, the in vivo contribution of CSCs to tissue repair remains elusive, partly because of the heterogeneity of the cardiac stem cell populations identified so far. The broad diversity of cardiac stem cells in terms of the described markers and properties suggests the existence of a wide range of different adult cardiac stem cell populations [21,22,23]. From a developmental point of view, the formation of the human heart is a complex process that is still not fully understood. A primary heart tube is formed out of the cardiogenic plates from the anterior splanchnic mesoderm after the third week of embryogenesis [24]. After a rightward looping process, the septation process follows. Here, primitive chambers are subdivided to form the cardiac atria and ventricles [24]. During septation of the outflow tract of the ventricle, the aortic orifice gets in contact with the left ventricle while the pulmonary orifice remains situated above the right ventricle. During this process, cardiac neural crest cells enter the outflow tract as condensed mesenchyme between the aortic and pulmonary orifice [24,25,26]. The neural crest is a transient embryonic structure that was initially described by Wilhelm His in the development of the chick embryo as the intermediate chord appearing between the neural chord and the future ectoderm [27]. During embryonic development, neural crest stem cells migrate and give rise to a broad range of tissues including the heart, where neural crest cells populate the myocardium and contribute to myocardialization, although most of these cells disappear in later stages upon apoptosis [24,26]. However, neural crest-derived populations of cardiac stem cells have recently been described in the adult hearts of mice and zebrafishes [28,29,30,31,32]. In particular, El-Helou and colleagues demonstrated the presence of NCSCs in the adult rat heart via expression of Nestin [31], a characteristic NCSC-marker associated with proper self-renewal of stem cells [33,34], while Tomita and coworkers could show that Nestin^+^ NCSCs in the mouse heart give rise to cardiomyocytes in vivo [35]. Furthermore, human congenital heart diseases like DiGeorge Syndrome, CHARGE Syndrome and Alagille Syndrome were linked to defective cardiac neural crest function [36,37,38], suggesting a possible contribution of neural crest-derived cardiac stem cells to normal cardiac regeneration. However, neural crest-derived stem cell populations in the human heart have not been identified so far.

Within this study, we directly compared Nestin^+^/CD105^+^ adult hCSCs to neural crest-derived ITSCs from the human nasal cavity using global gene expression profiling. Next to other NCSC-pools, ITSCs were reported to be positive for Nestin, S100 and p75 on the protein level and showed the ability to give rise to ectodermal as well as mesodermal cell types in vitro and in vivo [8,39,40,41,42]. Likewise, we detected the expression of the proteins Nestin, S100 and p75 in cultured hCSCs, suggesting a potential relation of hCSCs to the neural crest. Our comparison of both stem cell populations using bioinformatic tools led to the identification of 4367 differentially expressed genes (DEGs), while respective identified GO-terms of differential gene expression were associated with the tissues of origin, namely the heart and the olfactory or respiratory epithelium of the nose. Interestingly, a broad range of neural crest-associated genes was found to be expressed in both stem cell populations. We further compared the global gene expression profiles of hCSCs and ITSCs with published RNA-Seq data of adipose-derived mesenchymal stem cells (AdMSCs), CD34^+^ hematopoietic stem cells (HSCs) [43] and cardiosphere-derived cells (CDCs) [44]. In comparison to hematopoietic stem cells, stem cell-associated GO-terms like ’tissue morphogenesis’, ’vasculature development’ or ’embryonic development’ were upregulated in hCSCs and ITSCs, which may hint to a shared regulation of their stem cell properties. In addition, our findings may help to understand the underlying molecular kinetics determining the differences between various adult human stem cell populations.

## 2. Materials and Methods

### 2.1. Cell Isolation and Cultivation

Human cardiac stem cells were isolated and cultivated as previously described [17] according to local and international guidelines (declaration of Helsinki) after informed and written consent. Isolation and further experimental procedures were ethically approved by the ethics commission of the Ruhr-University Bochum (Faculty of Medicine, located in Bad Oeynhausen) (approval reference number eP-2016-148).

Human inferior turbinate stem cells were isolated and cultivated after informed written consent according to local and international guidelines (declaration of Helsinki) as previously described [8,45]. Isolation and further experimental procedures were ethically approved by the ethics commission of the Ärztekammer Westfalen-Lippe and the medical faculty of the Westfälische Wilhelms-Universität (Münster, Germany) (approval reference number 2012-15-fS).

### 2.2. Lentiviral Transduction of hCSCs

HCSCs were transduced by lentivirus with the cFUG-W plasmid. Lentivirus production was carried out in HEK293 cells with packaging plasmid Δ8.91, VSV-G envelope plasmid and cFUG-W transfer vector by calcium-phosphate precipitation. Δ8.91 and VSV-G were gifts from David Baltimore [46]. Supernatant was harvested 48 h after transfection and lentivirus was concentrated by ultracentrifugation (50,000× *g*, 4 °C, 2 h).

### 2.3. Coculture of GFP-hCSCs and Primary Mouse Cardiomyocytes

Primary mouse cardiomyocytes were isolated from newborn mice according to Streejt and colleagues [47]. Prior to coculture, mouse cardiomyocytes were treated with 10 µg/mL Mitomycin C (Sigma Aldrich) according to the manufacturer’s instructions. Coculture with hCSCs was carried out in DMEM-F12 with 5% horse serum (Dianova).

### 2.4. Immunohistochemistry and Immunocytochemistry

Cultivated cells were fixed for 20 min using 4% paraformaldehyde (PFA), washed and permeabilized in PBS with 0.02% TritonX-100 (Sigma Aldrich) and supplemented with 5% goat serum for 30 min. The applied primary antibodies were diluted in PBS as followed: rabbit anti-Nestin 1:200 (Millipore), mouse anti-S100B 1:500 (Sigma Aldrich), rabbit anti-Slug 1:100 (Cell-Signaling Technology), rabbit anti-p75 1:500 (Cell-Signaling Technology), mouse anti-β-III-tubulin 1:100 (Promega), rabbit anti-neurofilament-L 1:50 (Cell-Signaling Technology), anti-vGlut (Millipore) and anti-Synaptophysin (Millipore). They were applied for 1 h (cells) at room temperature. After three washing steps, secondary fluorochrome-conjugated antibodies (Alexa 555 anti-mouse or Alexa 488 anti-rabbit, Invitrogen, Life Technologies GmbH) were applied for 1 h at RT with a dilution ratio of 1:300. Nuclear staining was realized by incubation with 4,6-Diamidin-2-phenylindol (DAPI) (1 μg/mL, Applichem) in PBS for 15 min at RT. Finally, the samples were mounted with Mowiol (self-made). Imaging was performed using a confocal laser scanning microscope (CLSM 780, Carl Zeiss) and image processing was executed with ImageJ and CorelDRAW [48] (open source and Corel Corporation).

### 2.5. Induced Neuronal Differentiation

Neuronal differentiation in the isolated cells was induced following the protocol described by Müller and colleagues [39]. Briefly, cells were seeded with a density of 2 × 10^5^ cells per 6-well in hCSC-medium. After 48 h, neural differentiation was induced with a neuronal induction medium containing 1 μM dexamethasone (Sigma Aldrich), 2 μM insulin (Sigma Aldrich), 500 μM 3-isobutyl-1-methylxanthine (Sigma Aldrich) and 200 μM indomethacin (Sigma Aldrich). Cells were fed every 2–3 days by removing half of the medium and adding the same amount of fresh prewarmed medium. After 7 days of culture, maturation of the cells was induced by adding retinoic acid (0.5 mM) (Sigma Aldrich) and N2-supplement (1x) (Gibco) over 2 days. Afterwards, retinoic acid was removed while N2 was applied until neuronal maturation at day 28. As undifferentiated control, cells were cultured in hCSC-medium as described above. After 28 days, the protein expression was analyzed by immunocytochemical staining.

### 2.6. Osteogenic Differentiation of hCSCs

The osteogenic differentiation of hCSCs was induced by biochemical cues according to Greiner and coworkers [45]. Briefly, cells were seeded in hCSC-medium at a density of 3 × 10^3^ cells/cm^2^. After 48 h the medium was switched to an osteogenic induction medium supplemented with 100 nM dexamethasone (Sigma Aldrich), 0.05 mM L-ascorbic acid-2-phosphate (Sigma Aldrich) and 10 mM β-glycerophosphate (Sigma Aldrich). The medium was changed every 2–3 days. After 21 days, differentiated cells were processed for RNA-Isolation as described below. For undifferentiated controls, cells were cultured in hCSC-medium as described above.

### 2.7. Adipogenic Differentiation of hCSCs

For adipogenic differentiation, hCSCs were cultivated in DMEM (Sigma Aldrich) containing 10% FCS (Sigma Aldrich) and plated at a density of 4 × 10³ cells /cm². After 48 h, 1 μM dexamethasone (Sigma Aldrich), 2 μM insulin (Sigma Aldrich), 500 μM 3-isobuthyl-1-methylxanthine (Sigma Aldrich) and 200 μM indomethacin (Sigma Aldrich) were added to the medium and cultivated for 72 h. Afterwards, the medium was switched and cells were cultivated for 4 days in DMEM containing 10% FCS and 2 μM insulin (Sigma Aldrich) to induce adipogenic differentiation. These two media were alternatingly used and changed every 4 days for 3 weeks. As undifferentiated control, cells were cultured in hCSC-medium as described above.

### 2.8. Quantitative PCR

The RNA isolation was performed using the NucleoSpin RNA Kit (Macherey Nagel, Bethlehem, PA, USA) according to the manufacturer’s guidelines. The quality and concentration of the obtained RNA was quantified by a spectrophotometer (Thermo Fisher Scientific, Waltham, MA, USA). For cDNA synthesis, the First Strand cDNA Synthesis Kit (Thermo Fisher Scientific) was applied in accordance with the manufacturer’s guidelines. qPCR was carried out using Perfecta SYBR green Supermix (quantaBio, Beverly, MA, USA) following the manufacturer’s instructions with primers for *PPARG* (fwd: GGATGCAAGGGTTTCTTCCG, rev: AACAGCTTCTCCTTCTCGGC), *ON* (fwd: AAACATGGCAAGGTGTGTGA, rev: TGCATGGTCCGATGTAGTC) and *GAPDH* (fwd: CATGAGAAGTATGACAACAGCCT, rev: AGTCCTTCCACGATACCAAAGT).

### 2.9. RNA-Seq and Bioinformatic Analysis

RNA of cultured cells was isolated with the NucleoSpin RNA Kit (Macherey Nagel, Düren, Germany) and stabilized with RNAstable (Biomatrica, San Diego, CA, USA) for transport at room temperature. RNA was sequenced by Novogene (Beijing, China) using the Illumina Hiseq4000 platform with a paired end 150 bp strategy. RNA-Seq raw data are accessible at NCBI Gene Expression Omnibus with the accession number GSE129547. More data were downloaded from the NCBI Sequence Read Archive (SRA) with the accession numbers GSE140385 (CD34^+^ hematopoietic stem cells [43]), GSE142831 (adipose-derived mesenchymal stem cells) and GSE81827 (cardiosphere-derived cells [44]). Here, we took care to select datasets of paired end sequencing runs from the Illumina platform to minimize technical variability between the groups. From these studies, we selected the datasets of the control groups, to use only expression data of untreated cells. First, all data were processed in the same way: FastqQC (Version 0.11.19) was used for a first quality control of the raw data. Subsequently, trimming of low-quality bases and adapter clipping was performed with Trimmomatic-0.38 [49] with the following settings: PE; -phred33; ILLUMINACLIP:TruSeq3-PE.fa:2:30:10; LEADING:6; TRAILING:6; SLIDINGWINDOW:4:15; MINLEN:36. Clean reads were aligned to the *Homo sapiens* reference genome sequence (GRCh38) using STAR 2.7.3a [50] with the following parameters: runThreadN 8; limitBAMsortRAM 32000000000; --outBAMsortingThreadN 8; --outSAMtype BAM SortedByCoordinate; --outFilterMismatchNoverLmax 0.05; --outFilterMatchNminOverLread 0.8. FeatureCounts (version 2.0.0) was used to quantify the read number after mapping [51] with the following parameters: -T 4; -t gene; -g gene_id; -a Homo_sapiens.GRCh38.78.gtf. Differential gene expression analysis between two groups was performed using the DESeq2 R package [52]. Here, a publicly available script from Stephen Turner was used with slight modifications (https://gist.github.com/stephenturner/f60c1934405c127f09a6). GO-term enrichment and KEGG pathways analysis were performed using the gage package in R [53]. Here, a publicly available script from Stephen Turner was used with slight modifications (https://www.r-bloggers.com/2015/12/tutorial-rna-seq-differential-expression-pathway-analysis-with-sailfish-deseq2-gage-and-pathview/). The corresponding scripts are provided in the supplementary materials. Visualization of significantly enriched terms was performed using Graph Pad Prism 8.

## 3. Results

### 3.1. hCSCs Show a NCSC-Like Expression Pattern and Differentiate into Mesodermal and Ectodermal Derivates

**Figure 1:**
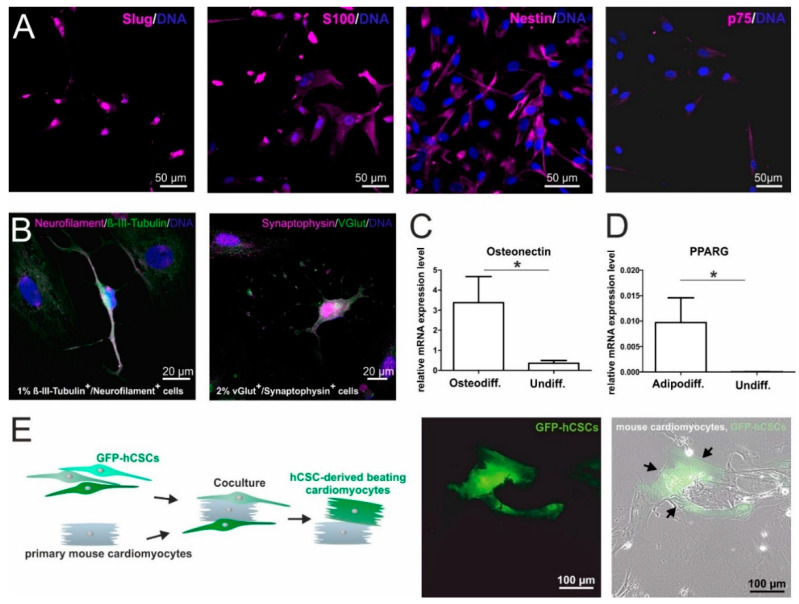
In vitro characterization of human cardiac stem cells (hCSCs): (**A**) Immunocytochemical stainings of cultured hCSCs showed the expression of the NCSC markers Slug, S100, Nestin and p75. (**B**) Directed differentiation of hCSCs generated Neurofilament^+^/β-II-Tubulin^+^ and Synaptophysin^+^/VGlut^+^ neuron-like cells. (**C**) Application of an osteogenic differentiation medium resulted in the upregulation of Osteonectin mRNA (Mann Whitney Test, * *p* < 0.05). (**D**) Application of an adipogenic differentiation protocol resulted in the upregulation of PPARG mRNA (Mann Whitney Test, * *p* < 0.05). (**E**) GFP^+^ hCSCs differentiate into beating cardiomyocytes (arrowheads) upon coculture with primary mouse cardiomyocytes.

For an initial comparison of hCSCs and ITSCs, we aimed to compare the marker expressions of hCSCs and ITSCs on the protein level in vitro. In a previous publication, we already showed that ITSCs express the neural crest-related stem cell markers Slug, S100, Nestin and p75 [8]. To investigate, whether hCSCs share this marker expression profile, we performed immunocytochemical stainings of cultured hCSCs and observed the presence of Slug, S100, Nestin and a slight expression of p75 proteins (Figure 1A). Notably, Slug protein seemed to be localized in the nuclear compartment, indicating its activity as a transcription factor (Figure 1A). In addition to the NCSC-like marker expression, we investigated the differentiation capacity of hCSCs in comparison to ITSCs. As already shown in a broad range of studies, ITSCs are able to differentiate very efficiently into mesodermal as well as ectodermal derivates like neurons, osteoblasts and adipocytes [8,39,40,54]. We applied these established protocols to hCSCs and detected 1–2% neuron-shaped cells positive for the neuronal markers Neurofilament, β-III-Tubulin, Synaptophysin and VGlut (Figure 1B). After directed osteogenic differentiation of hCSCs, osteonectin expression was significantly upregulated (Mann Whitney Test, *p* < 0.05) compared to an undifferentiated control (Figure 1C). HCSCs likewise successfully underwent adipogenic differentiation resulting in the significant upregulation (Mann Whitney Test, *p* < 0.05) of PPARG mRNA (Figure 1D) compared to undifferentiated controls. To investigate the differentiation of hCSCs into cardiomyocytes within a cardiomyogenic environment, we performed coculture experiments with primary neonatal beating mouse cardiomyocytes and lentiviral transduced GFP^+^ hCSCs. After 11 days of coculture, we detected GFP^+^ beating human cardiomyocytes next to mouse cardiomyocytes (Figure 1E, arrowheads). These observations confirmed the functionality of hCSC-derived cardiomyocytes. Notably, we already could show that hCSCs express common cardiomyocyte markers like α-actinin and Connexin43 after differentiation with biochemical cues [17]. In summary, hCSCs and ITSCs shared high similarities in the presence of marker proteins, whereas differentiation capabilities differed in dependence on the respective niche of the stem cell population (Figure S1).

### 3.2. Differential Gene Expression Between hCSCs and ITSCs Reflects the Particular Niches of Origin

**Figure 2:**
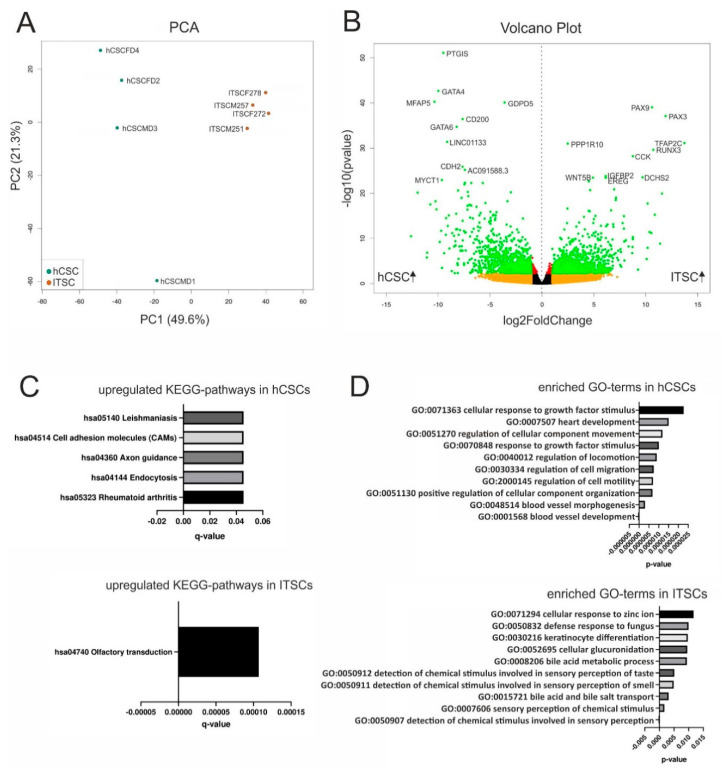
Differential gene expression between hCSCs and inferior turbinate stem cells (ITSCs). (**A**) Principal component analysis shows that hCSCs and ITSCs build separate clusters. (**B**) The volcano plot shows 4367 significantly DEGs (green dots). (**C**) KEGG pathway analysis reveals six pathways to be significantly (*p* < 0.05) up- or downregulated between hCSCs and ITSCs. (**D**) Top ten of the most significantly enriched GO-terms referring to biological processes.

We next extended our comparison of hCSCs and ITSCs from marker protein expression and differentiation capacities to global gene expression profiles by performing RNA-Seq of hCSCs from 4 distinct donors as well as of ITSCs from 4 different donors. A principal component analysis (PCA) showed that hCSCs and ITSCs formed distinct clusters, with PC1 explaining 49.6% of the total variance. Further, the gene expression patterns of the single hCSC donors seemed to be more heterogeneous than within ITSCs, visible by the distribution along the PC2 axis explaining 21.3% of the variance (Figure 2A). To investigate these differences in more detail, we visualized the DEGs between both groups in a volcano plot (Figure 2B). In total, 4367 genes were significantly differentially expressed (*p* < 0.05) with 2074 significantly upregulated in hCSCs (*p* < 0.05) and 2,293 significantly upregulated in ITSCs (*p* < 0.05). Interestingly, we found the genes for the transcription factors PAX3 and PAX9 to be significantly overexpressed in ITSCs (PAX3 *p* ≈ 7.9 × 10^−38^; PAX9 *p* ≈ 9.1 × 10^−40^). We next reduced data dimensionality by applying a KEGG pathway analysis. Here, five KEGG pathways were significantly (*q* < 0.05) upregulated in hCSCs compared to ITSCs while only the KEGG pathway hsa04740 ’olfactory transduction’ was significantly (*q* ≈ 1.1 × 10^−4^) upregulated in ITSCs compared to hCSCs (Figure 2C). This may reflect the origin of the examined cell populations, however the enrichment of only six significantly up- or down regulated KEGG pathways could also demonstrate that this analysis was not appropriate to visualize the differences between hCSCs and ITSCs. We therefore analyzed the GO-term enrichment of biological processes. Among the top ten of the most significantly enriched GO-terms of genes upregulated in hCSCs, we found terms associated with cardiovascular development like ’blood vessel development’ (*p* ≈ 1.9 × 10^−7^), ’blood vessel morphogenesis’ (*p* ≈ 3 × 10^−6^) and ’heart development’ (*p* ≈ 1.5 × 10^−5^) (Figure 2D). Furthermore, the top ten of the most significantly enriched GO-terms of genes upregulated in ITSCs comprised terms like ’detection of chemical stimulus involved in sensory perception’ (*p* ≈ 1.5 × 10^−4^), ’sensory perception of chemical stimulus’ (*p* ≈ 1.7 × 10^−3^) and ’detection of chemical stimulus involved in sensory perception of smell/taste’ (*p* ≈ 4.9 × 10^−3^ and *p* ≈ 5.1 × 10^−3^) (Figure 2D). Since hCSCs were derived from the left atrial appendage of the human heart and ITSCs were located in the inferior turbinate of the nose, these GO-terms were clearly linked to the tissue of origin of the examined stem cell populations. Remarkably, general stem cell-associated GO-terms of biological processes were not enriched when comparing global gene expression profiles of hCSCs and ITSCs, leading to the assumption that both cell populations did not differ significantly in their stem cell marker expression profiles. Further, we detected several markers for neural crest-derived stem cells [55] that were expressed in both stem cells populations (Table 1).

To further elucidate a potential contribution of neural crest-derived cells to adult cardiac structures and cardiac functionality, we carefully reviewed literature reporting mutations in known neural crest-associated genes expressed in ITSC and in hCSCs (Table 1). Interestingly, a wide range of the resulting defects is represented by craniofacial abnormalities as well as congenital cardiac defects such as Baraitser–Winter syndrome, oculodentodigital dysplasia, Pallister-Hall syndromes (PHS), Alagille syndrome, Autosomal dominant form of Adams-Oliver syndrome, Hajdu Cheney Syndrome (HCS), Hirschsprung’s disease, Loeys–Dietz syndrome, Saethre-Chotzen syndrome, and Kabuki syndrome.

### 3.3. hCSCs and ITSCs Share Higher Similarities in Gene Expression Profiles with AdMSCs and CDCs than with HSCs

**Figure 3:**
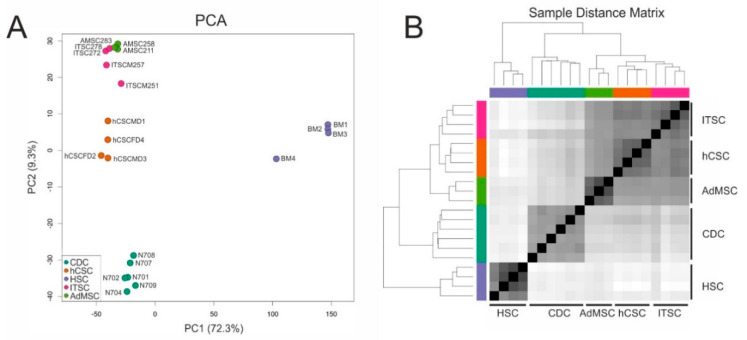
Comparison of global gene expression profiles of adult stem cell populations from different sources. (**A**) Principal component analysis reveals that all cell populations cluster independently. (**B**) The sample distance matrix reveals great differences between hematopoietic stem cells (HSCs) and other adult stem cell populations.

A comparison of two human stem cell populations from the adult heart (hCSCs) and the adult inferior turbinate of the nose (ITSCs) in terms of gene or protein expression as well as differentiation capacities showed differences that clearly reflected the niches or tissues of origin but no germ layer-associated differences. Therefore, we were interested in a comparison of hCSCs and ITSCs with other adult stem cell populations that were isolated and characterized independently in other labs. To compare more adult human stem cell populations on a global gene expression level, we accessed published RNA-Seq data of known human stem cell pools. Here, we took care to select datasets of paired end sequencing runs from the Illumina platform to minimize technical variability between the groups. In detail, we accessed expression data of cardiosphere-derived cells (CDC) [44], CD34+ hematopoietic stem cells (HSC) [43] and adipose-derived mesenchymal stem cells (AdMSC) (NCBI GEO-accession number GSE142831). From these studies, we selected the datasets of the control groups, to use only expression data of untreated cells. First, all data were processed in the same way: Trimming of raw reads was performed with Trimmomatic-0.38 [49] to clip adapter sequences and low-quality bases. Subsequently, clean reads were mapped to the reference genome sequence GRCh38 using STAR-2.7.3a [50] and read counts were quantified with featureCounts [51]. The resulting data were further analyzed with the DESeq2 pipeline. Principal component analysis (PCA) revealed that all cell populations formed individual clusters along the PC1 and PC2 axes. The greatest differences existed between HSCs and all of the other stem cell populations, as PC1 explains 72.3% of the variance. However, PC2 also distributed the stem cell populations with 9.3% of the variance (Figure 3A). Interestingly, on the PC2 axis, adipose-derived MSCs and ITSCs were closer to each other than to hCSCs. 

**Figure 4:**
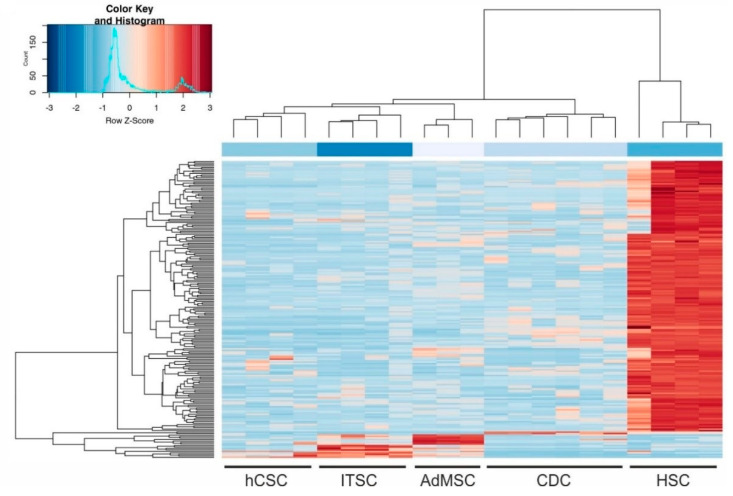
Hierarchically clustered heatmap of the 200 genes with the highest variance among all samples.

We could further confirm that the global transcriptome of cardiosphere-derived cells sequenced by Harvey and colleagues [44] shows higher similarity to, and is closer to hCSCs than to any of the other adult stem cell population. These observations indicate a shared heart stem cell-specific expression profile of hCSCs and CDCs and may allow the conclusion that minor differences visible on the PC2 axis were related to the niches of the stem cell populations. The large differences between HSCs and the other examined stem cell populations were also visible in a sample distance matrix (Figure 3B) and a hierarchical clustered heatmap of the 200 genes with the highest variance among all samples (Figure 4). Here, all cell populations clustered individually, but CS were more distinct from ITSCs, hCSCs and AdMSCs while HSCs showed the greatest differences to the other populations. A detailed list of all 200 genes is provided in Table S1.

### 3.4. hCSCs and ITSCs Overexpress Stem-Cell Associated Genes When Compared with HSCs

**Figure 5:**
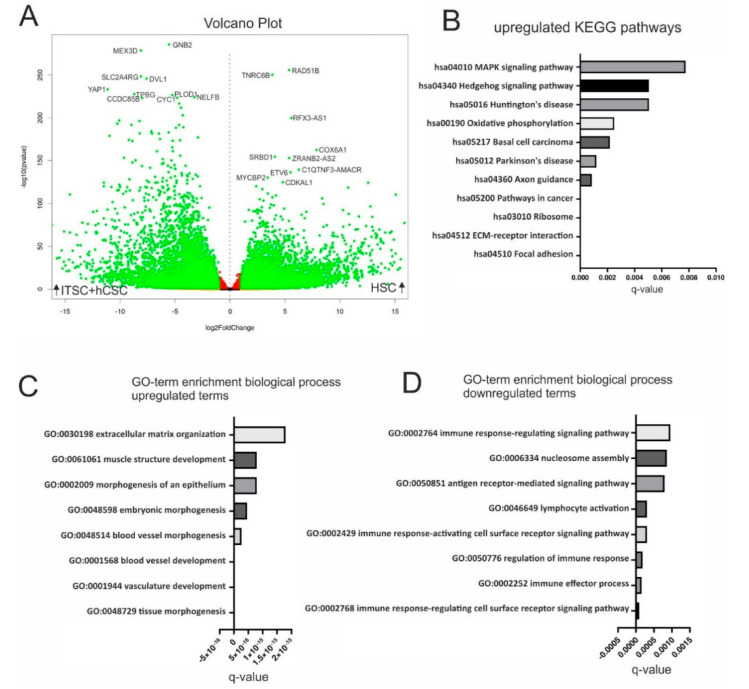
Comparison of ITSCs and hCSCs with HSCs by differential gene expression analysis. (**A**) Volcano plot shows 16,129 genes to be significantly (*q* < 0.05) differentially expressed (green dots) in hCSCs and ITSCs compared to HSCs. (**B**) Top ten of the most significantly upregulated KEGG pathways in hCSCs and ITSCs (*q* < 0.05). (**C**) Top ten of the most significantly enriched GO-terms in hCSCs and ITSCs (*q* < 0.05). (**D**) Top ten of the most significantly enriched GO-terms in HSCs (*q* < 0.05).

Based on the observation that hCSCs and ITSCs shared highly similar global gene expression patterns with AdMSCs and CDCs but not with HSCs, we decided to compare the transcriptomic profiles of hCSCs and ITSCs with HSCs in more detail. We therefore examined differential gene expression between the datasets of hCSCs and ITSCs compared to HSCs. A volcano plot demonstrated the significant upregulation of 7154 (*p* < 0.05) genes in hCSCs and ITSCs compared to HSCs while 8975 genes were significantly downregulated (*p* < 0.05) in this comparison (Figure 5A). We further conducted a KEGG pathway analysis. Here, we calculated overrepresented KEGG pathways and plotted the ten most significantly enriched pathways (q < 0.05). Here, the upregulated KEGG pathways with the highest significance were ’Focal adhesion’ (q ≈ 3.5 × 10^−7^) and ’ECM-receptor interaction’ (q ≈ 7.4 × 10^−6^) (Figure 5B). Both pathways describe the adherent character of hCSCs and ITSCs either to a cell culture surface or, when grown as spheres, to other cells. Interestingly, the KEGG pathway MAPK was significantly enriched in genes that were upregulated in hCSCs and ITSCs compared to HSCs (Figure 5B). We further performed GO-term enrichment of DEGs in hCSCs and ITSCs in comparison to HSCs. The GO-terms corresponding to biological processes revealed high enrichment in stem cell- and tissue repair-associated terms like ’tissue morphogenesis’ (q ≈ 1 × 10^−19^), ’vasculature development’ (q ≈ 2.1 × 10^−19^), ’blood vessel development’ (q ≈ 2 × 10^−18^) and ’embryonic morphogenesis’ (q ≈ 4.5 × 10^−16^) (Figure 5C). In contrast, the GO-terms ’Immune response-regulating cell surface receptor signaling pathway’ (q ≈ 9.1 × 10^−5^), ’immune effector process’ (q ≈ 1.5 × 10^−4^) and ’regulation of immune response’ (q ≈ 1.8 × 10^−4^) were significantly downregulated in hCSCs and ITSCs compared to HSCs (Figure 5D). These data demonstrates that hCSCs and ITSCs share a transcriptional profile that is associated with stem cell properties like tissue morphogenesis, vasculature development and embryonic morphogenesis, while HSCs highly overexpress genes that are related to immune response mechanisms.

## 4. Discussion

The present study describes the side-by-side comparison of two different adult human stem cell populations from the left atrial appendage of the heart and from the inferior turbinate of the nose, based on in silico and in vitro data. We detected stem cell marker proteins like Nestin, p75 and S100 in both stem cell populations, while global gene expression data revealed significant differences between the populations reflecting the particular niches of origin.

Most studies investigating cardiac stem cells focus on their potential to differentiate into cardiogenic cell types like cardiomyocytes, endothelial cells and smooth muscle cells [18,19,44,97], mainly with regard to a potential use in regenerative medicine. In general, the description of these cell populations focuses on the expression of cell surface markers like c-kit or Sca1—commonly accepted markers of cardiac stem cells. We very recently isolated a population of Sca1^+^/cKit^−^ cardiac stem cells from the adult human heart (hCSCs), which were able to give rise to α-actinin/Connexin43-positive cardiomyocytes after directed differentiation in vitro [17]. In accordance with our previous findings, hCSCs successfully differentiated into beating cardiomyocytes after exposure to a cardiomyogenic environment in the present study. Next to differentiation into cardiomyocytes, we already observed the presence of the intermediate filament Nestin in distinct spots in heart auricle tissue on protein level [17]. Within this study, we further extended these findings by the detection of Nestin also in cultured hCSCs. In the murine system, Nestin^+^ stem cells can be derived from the adult heart that give rise to neurons [31,35,98], however a neurogenic differentiation potential in human adult cardiac stem cells has not been shown so far. Here, we observed a small proportion of 1% Neurofilament^+^/β-III-Tubulin^+^ and 2% Synaptophysin^+^/VGlut^+^ cells with neuron-like shape that were derived from human cardiac stem cells upon application of a defined medium. We likewise detected a shared marker expression profile of undifferentiated hCSCs with ITSCs, a known neural-crest derived adult stem cell population [8]. In addition to Nestin, the neural crest stem cell markers p75, Slug and S100 were also present in both cell populations on protein level. 

The neural crest was initially described by Wilhelm His in the development of the chick embryo as the intermediate chord appearing between the neural chord and the future ectoderm [27]. After neurulation, neural crest cells migrate to a broad range of target tissues within the developing organism and give rise to various cell populations—cells of mesodermal and ectodermal type [33]. In addition, neural crest-derived cells also persist as adult stem cell populations within the adult body [8,9,99,100,101]. Mutations in neural crest-related genes result in severe developmental defects that are often presented in symptoms like malformations of craniofacial tissues, but also cardiac defects. Here, we provide a list of syndromes and diseases that are caused by mutations of neural crest associated genes which are expressed in ITSCs as well as in hCSCs. Notably, ten of these defects result in craniofacial malformations and also inherited heart defects (Baraitser–Winter syndrome [56], oculodentodigital dysplasia [61,62], Pallister-Hall syndromes (PHS) [65], Alagille syndrome [75], Autosomal dominant form of Adams-Oliver syndrome [73,74], Hajdu Cheney Syndrome [76,77], Hirschsprung’s disease [81,82], Loeys–Dietz syndrome [85], Saethre-Chotzen syndrome [92,93], and Kabuki syndrome [94,95,96]). Although a range of stem cell populations was described to be present in the human heart [6,102,103], a potential relation of these human cardiac stem cell populations to the neural crest has not been described so far. Accordingly, a potential developmental relation of cardiac stem cells to the neural crest has already been suggested in mice and rats [28,29,31,32,104]. In particular, El-Helou and colleagues demonstrated the presence of NCSCs in the adult rat heart via expression of Nestin [31], a characteristic NCSC-marker associated with proper self-renewal of stem cells [33,34], while Tomita and coworkers showed that Nestin^+^ NCSCs in the mouse heart give rise to cardiomyocytes in vivo [35]. Notably, similar to neural crest-derived stem cell populations residing within the head and neck region [8,9,39,105,106], hCSCs exhibited an extended differentiation capability by giving rise to cardiomyocytes, but also other mesodermal and ectodermal cell types. However, we also observed differences in the differentiation potential of both stem cell populations particularly regarding the extraordinary high differentiation capability of ITSCs into the neuronal lineage (70%) [39,40,41], which is only minor in hCSCs (1–2%). We suggest these differences to depend on the niches of the stem cell populations, which is in line with the GO-term enrichment analysis of DEGs in hCSCs and ITSCs.

As already postulated by Iancu and colleagues, not only the presence of single cell surface markers but rather an extensive marker profile, consisting of a combination of specific cell surface markers and global gene expression, are required to distinguish distinct cardiac and non-cardiac stem cell populations [107]. In this regard, we performed RNA-Seq to compare global transcriptional profiles of hCSCs with ITSCs. In a previous study, we showed a large difference in global gene expression between ITSCs and human embryonic stem cells using microarrays [8]. Here, RNA-Seq allowed us to detect 4367 genes that were significantly differentially expressed between hCSCs and ITSCs. Although both cell populations formed distinct clusters in a principal component analysis, the most significantly enriched GO-terms only comprised a description of the particular tissues of origin. Interestingly, GO-terms that are linked to certain germ layers of origin are not enriched among the DEGs, leading to the conclusion that hCSCs and ITSCs may share a joint developmental origin. To the best of our knowledge, analyses of RNA-Seq data comparing different stem cell populations from different laboratories are rare. Jansen and colleagues compared different mesenchymal stem cell populations side-by-side in a microarray experiment and could show that the functional status of cell populations can indeed be monitored by the transcriptomic profile [108]. However, the usability of global transcriptome data for identification of a cell population and its functional status also implies the limitation that not every mRNA is translated into a functional protein. Using next-generation sequencing, Taskiran and coworkers compared human bone marrow mesenchymal stem cells and dermal fibroblasts and identified several homeobox genes to be differentially expressed [109]. Paired box (PAX) genes are suggested to contain homeobox genes. We likewise detected the PAX genes PAX3 and PAX9 to be differentially expressed between hCSCs and ITSCs. PAX3 is known as key player in cranial neural crest development and is associated with neural crest-related diseases like Waardenburg syndrome [110], while PAX9 initiates tooth development [15,111,112,113]. 

In addition to the side-by-side analysis of hCSCs and ITSCs, we compared here for the first time the global gene expression profiles of five adult human stem cell populations from diverse niches. The principal component analysis provided here demonstrates that hCSCs, ITSCs, AdMSCs and CDCs share much more similarities with each other than with HSCs. Interestingly, adipose-derived MSCs and ITSCs were closer to each other than to hCSCs on the PC2 axis, suggesting the observed variances resulted from biological differences between the different stem cell populations rather than from the use of different library preparation protocols or sequencing platforms. The MSC population in this study is derived from the adipose tissue while HSCs are isolated from the bone marrow. However, during development, both stem cell populations have their origin in the mesoderm. Remarkably, although HSCs and MSCs share their developmental origin in the mesoderm, their transcriptomic profiles show great differences in our analysis. Likewise, regarding differences between HSCs and MSCs, 16,129 genes were found to be differentially expressed between hCSCs and ITSCs compared to HSCs while a direct comparison of hCSCs and ITSCs revealed only 4367 DEGs. GO-term analysis demonstrated the enrichment of stem cell-associated terms like ’tissue morphogenesis’, ’vasculature development’, ’blood vessel development’ and ’embryonic morphogenesis’, while terms like ’immune response-regulating cell surface receptor signaling pathway’, ’immune effector process’ and ’regulation of immune response’ were significantly downregulated in hCSCs and ITSCs compared to HSCs. This may indicate a similar regulation of stem cell-associated transcripts in hCSCs and ITSCs. Furthermore, our data reflect the diverging developmental potentials of HSCs and other more tissue-bound stem cell populations. The upregulation of immune-regulatory pathways and GO-terms is a known characteristic of hematopoietic stem- and progenitor cells and reflects the hematopoietic fate of these cells [114,115,116]. The terms ’extracellular matrix organization’ and ’morphogenesis of an epithelium’ upregulated in hCSCs and ITSCs compared to HSCs underline the adherent character of non-HSC populations. This linkage is further emphasized by the upregulation of the terms ’focal adhesion’ and ’ECM-receptor interaction’ as most significantly upregulated KEGG-pathways in hCSCs and ITSCs. Interestingly, also the term ’MAPK signaling pathway’ was among these upregulated KEGG pathways. We already identified p38 MAPK as a crucial pathway mediating proliferation of blood serum-treated hCSCs [17]. The dimensionality of data that can be gained by RNA-Seq of cell populations is very large due to the high number of expressed genes. In our analysis, we detected 16,129 DEGs. This exceeds the number of proteins, which can be analyzed in comparative methods such as 2D gel or LC/MS analysis with 500–5000 proteins per sample [117,118,119]. However, single cell RNA-Seq (scRNA-Seq) might increase the amount of information that can be gained from such analyses. This would especially be useful for the examination of subtypes within a population [120]. In the context of the present study, we aimed to investigate the differences between distinct stem cell populations and therefore applied bulk RNA-Seq, which is a more robust and cost-effective method. Furthermore, the analysis of RNA is limited since not every expressed mRNA is related to a functional protein. The present study faces this challenge by providing both RNA-Seq data as well as a selection of stem cells markers on the protein level, accompanied by functional differentiation assays. In addition, future studies comparing the proteomes of adult stem cell populations may allow the transfer of our observations on the global transcriptome level to the functional protein level.

## 5. Conclusions

In summary, we provide a direct comparison of Nestin^+^/CD105^+^ adult hCSCs and neural crest-derived ITSCs from the human nasal cavity regarding the presence of molecular marker proteins, their differentiation capacities as well as their global transcriptomes. We show that transcriptional differences between hCSCs and ITSCs depend on their particular niches, which is also reflected on a functional level regarding their differentiation potentials. However, a potential difference in their developmental origins could not be found based on RNA-Seq data, while a broad range of neural crest-associated genes was found to be expressed in both stem cell populations, suggesting the neural crest as a developmental origin of hCSCs. We further extended these findings by the comparison of hCSCs and ITSCs with other known adult stem cell populations, identifying HSCs as a population with less stem cell-associated but more immune regulatory properties. The analyses provided here might help to understand the global transcriptional differences between different adult human stem cells populations, although our observations are limited to the mRNA level. 

## Figures and Tables

**Table 1:** Diseases related to mutations in neural crest-associated genes expressed in hCSCs and ITSCs. The read counts represent the mean of four replicates of hCSCs and ITSCs respectively. The mean read count among all samples and genes was 957.

Gene	Gene-ID	Name	Defects	Mean Read Counts	Ref.
*ACTB*	ENSG00000075624	Baraitser–Winter syndrome	defects in the development of the brain, eyes (colomba), and other facial structures. Other defects may present as short stature, ear anormalities with hearing loss, cardiac malformations, polydactyly, renal malformations, neurologic disorders.	hCSC: 186,465; ITSC: 201,934.5	[56]
*CTNNB1*	ENSG00000168036	Increased tumorigenicity	mutations of the *CTNNB1* gene are frequent (40–60% of cases) in endometrioid endometrial carcinoma (EEC) but have also been detected in a broad range of other cancer types.	hCSC: 19,904.75; ITSC: 20,509.5	[57,58]
*EDN1*	ENSG00000078401	Recessive Auriculocondylar syndrome	micrognathia, temporomandibular joint and condyle anomalies, microstomia, prominent cheeks, and question-mark ears (QMEs).	hCSC: 1471.75; ITSC: 34.5	[59]
*FZD7*	ENSG00000155760	Increased tumorigeneicity	upregulated in several cancer types including intestinal tumors, hepatocellular carcinomas, gastric cancer and breast cancer and is important for progression, invasion and metastasis.	hCSC: 5757.25; ITSC: 3631	[60]
*GJA1*	ENSG00000152661	Oculodentodigital Dysplasia	digital malformations, craniofacial anomalies, occasionally deafness and dysplasia of the ears, abnormal dentition, rarely cardiac abnormalities.	hCSC: 40,788.5; ITSC: 6454	[61,62]
*GLI3*	ENSG00000106571	Greig cephalopolysyndactyly (GCPS)	polydactyly, minor craniofacial abnormalities.	hCSC: 1482.75; ITSC: 1591.25	[63,64]
		Pallister-Hall syndromes (PHS)	hypothalamic hamartoma, polydactyly, dysplastic nails, rarely congenital heart defects.		[65]
*MSX1*	ENSG00000163132	Wolf–Hirschhorn syndrome	mental and growth retardation, craniofacial malformations, seizures, tooth agenesis.	hCSC: 399.25; ITSC: 1250	[66,67]
		Witkop syndrome	tooth agenesis, nail dysplasia.		[66,68]
		Non-syndromic orofacial clefts	cleft lip and/or cleft palate.		[66]
*NES*	ENSG00000132688	Development of the heart and brain	human nestin regulates cell proliferation in the heart and brain in a transgene mouse model.	hCSC: 7872; ITSC: 17,177.5	[69]
*NOTCH1*	ENSG00000148400	Alagille syndrome	intrahepatic bile duct paucity and cholestasis, cardiac malformations, ophthalmological abnormalities, skeletal anomalies, characteristic facial appearance, and renal and pancreatic abnormalities.	hCSC: 6108.25; ITSC: 2544.75	[70,71]
		Aortic valve disease	valve calcification.		[72]
		Autosomal dominant form of Adams-Oliver syndrome	terminal transverse limb malformations, an absence of skin, a partial absence of skull bones. Occasionally vascular anomalies, pulmonary or portal hypertension, retinal hypervascularization, congenital heart defects in 23% of the patients.		[73,74]
*NOTCH2*	ENSG00000134250	Alagille syndrome	See above.	hCSC: 27,494.5; ITSC: 11,911.25	[75]
		Hajdu Cheney Syndrome (HCS)	rare disease characterized by acroosteolysis, severe osteoporosis, short stature, craniofacial defects occasionally with cleft palate, wormian bones, neurological symptoms, sometimes cardiovascular defects.		[76,77]
*PAX3*	ENSG00000135903	Waardenburg syndrome	heterochromia, pigmentation anomalies, varying degrees of deafness.	hCSC: 1; ITSC: 3587.5	[78]
*PAX6*	ENSG00000007372	Aniridia	defects in the formation of the iris (absence or hypoplasia), cornea, lens, fovea, and optic nerve	hCSC: 44.75; ITSC: 42.5	[79]
*RET*	ENSG00000165731	medullary thyroid carcinoma	intermediate risk	hCSC: 53; ITSC: 82	[80]
		Hirschsprung’s disease	loss of neurons in the hindgut, congenital heart diseases (CHDs) are reported in 5% of the patients.		[81,82]
*SMAD2*	ENSG00000175387	Colorectal carcinoma	mutations in the tumor suppressors Smad2.	hCSC: 5217.25; ITSC: 5108	[83,84]
		Loeys–Dietz syndrome	defects in the connective tissue cause aortic aneurysms and arterial tortuosity, hypertelorism, and bifid/broad uvula or cleft palate.		[85]
*SNAI2*	ENSG00000019549	Increased tumorigeneicity	tumor growth and invasiveness in lung cancer, breast cancer progression, upregulated in colorectal carcinoma and may other cancer types.	hCSC: 3667; ITSC: 8666.75	[86,87,88,89]
*SNAI1*	ENSG00000124216	Increased tumorigeneicity	upregulation in breast cancer cells, ovarian cancer, and may other cancer types.	hCSC: 636.5; ITSC: 642.5	[88,90,91]
*TWIST*	ENSG00000122691	Saethre-Chotzen syndrome	craniofacial malformations, mild limb deformities, occasionally hearing loss, renal abnormalities and congenital heart malformations.	hCSC: 4361.75; ITSC: 8154.75	[92,93]
*KMT2D*	ENSG00000167548	Kabuki	craniofacial dysmorphism, minor skeletal anomalies, persistence of fetal fingertip pads, mild-to-moderate intellectual disability, and postnatal growth deficiency. Congenital heart defects in 70% of patients with mutations in the KMT2D gene.	hCSC: 7974.5; ITSC: 6808.75	[94,95,96]

## Supplementary Materials

The following are available online at https://www.mdpi.com/2079-7737/9/12/435/s1, Figure S1: Molecular characterization of ITSCs. (**A**) Cultured ITSCs express the neural crest stem cell markers S100 and Nestin. (**B**) Neurogenic differentiation potential of ITSCs, Table S1: Corresponding gene identities to the heatmap in Figure 4.
