# Supplementary figures and images for: Transcriptome Analysis Reveals High Similarities between Adult Human Cardiac Stem Cells and Neural Crest-Derived Stem Cells
Source: Biology (Basel). 2020 Dec 1;9(12):435. doi: 10.3390/biology9120435 (PMC7761507; doi:10.3390/biology9120435)

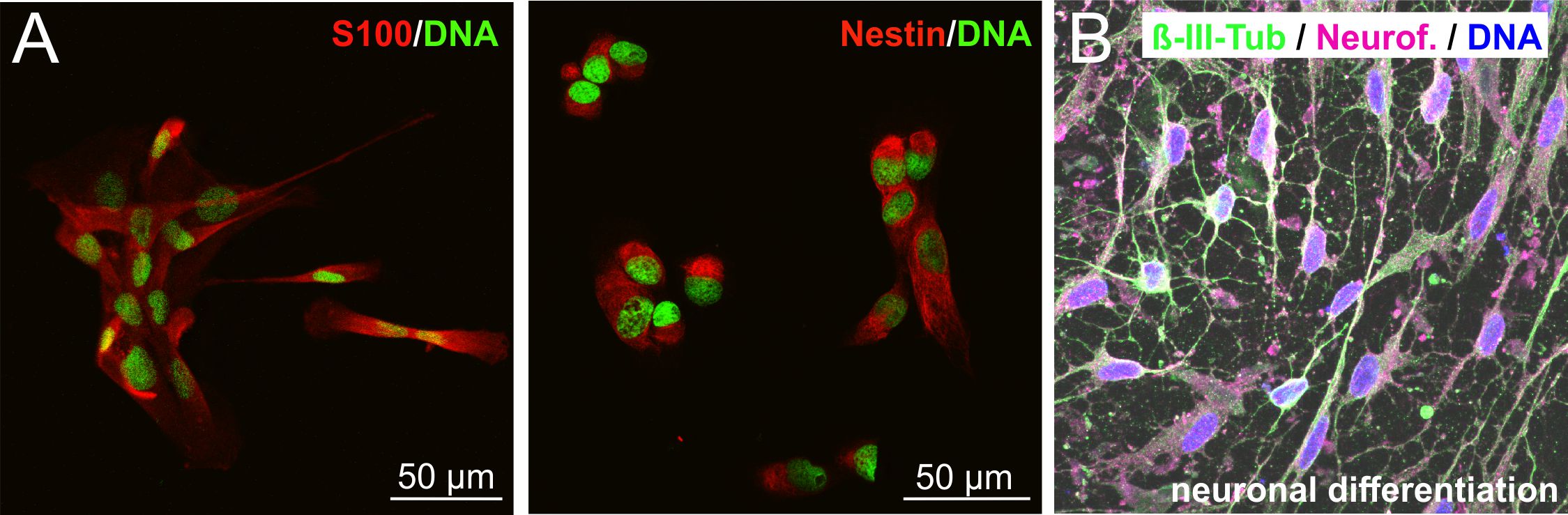

Supplement: Supplementary file 1 [file biology-09-00435-s001.zip › biology-1005219-suppl-final/Supplemental_material/Figure S1.jpg]
